# Dental College Association

**Published:** 1884-09

**Authors:** 


### Dental College Association.

We ask special attention to the proceedings of the Association of Dental College Faculties, organized at New York, August 4th. Quite an advanced step was there made in the matter of dental education. Ten colleges were fully represented, two or three others by letter. It is very desirable that all the colleges not there represented, and who wish or can consistently join the Association, will do so at once, as it will be seen, provision is made for these, and it is only necessary for any college wishing to become a member of the Association to signfy the fact to the officers, when the name will be placed on the roll of membership. Though everything was not done that the more progressive could wish, yet a very great advance was made by the organization of this society, and in the work done by it. It would seem as though no college could afford to stand independent of this movement. Dental education is largely in the hands of the colleges, and here, as elsewhere, "in union there is strength."

No one has anything to gain by standing outside, but much to lose. Probably no movement inaugurated for the last score of years has had the influence upon the profession that this will have.

The Associotion of State Boards of Dental Examiners met at Saratoga on the 8th of August. This body fully concurred in all that was done by the College Association, and the different Boards will doubtless give their influence to further the objects of the College Association.

